# Relevance of Visual Acuity Measurement for Therapeutic Decisions in Age-Related Macular Degeneration

**DOI:** 10.3390/jcm12020522

**Published:** 2023-01-09

**Authors:** Thibaud Mathis, Batoul El Ameen, Mayeul Chaperon, Yasmine Serrar, François Devin, Mikhail Dziadzko, Amina Rezkallah, Laurent Kodjikian

**Affiliations:** 1 Service d’Ophtalmologie, Hôpital Universitaire de la Croix-Rousse, Hospices Civils de Lyon, 69004 Lyon, France; 2 UMR5510 MATEIS, CNRS, INSA Lyon, Université Lyon 1, 69100 Villeurbanne, France; 3 Centre Monticelli Paradis, 13008 Marseille, France; 4 Groupe Almaviva Santé, Clinique Juge, 13008 Marseille, France; 5 U1290 RESHAPE, Université Claude Bernard Lyon 1, INSERM, 69004 Lyon, France

**Keywords:** age-related macular degeneration, intravitreal injection, optical coherence tomography, visual acuity

## Abstract

The aim of this study is to assess if the decision to retreat could be determined by anatomical criteria (mostly driven by optical coherence tomography (OCT)-guided strategy) rather than the gold standard (visual acuity (VA) and OCT) in patients with neovascular age-related macular degeneration (nAMD). A cross-sectional study of 142 eyes already treated for nAMD from September 2021 to December 2021 was performed. At inclusion, a first therapeutic decision was made based on the analysis of the OCT. This decision was then maintained or modified after being made aware of the patient’s VA. Sensitivity, specificity, positive predictive value (PPV) and negative predictive value (NPV) were calculated. The OCT-guided strategy matched the gold standard for treatment decisions in 131 of the 142 eyes included (92.3%). The sensitivity and specificity of the OCT-guided strategy for the retreatment decision were 94.0% and 89.8%, respectively. PPV and NPV were 92.9% and 91.4%, respectively. Considering the treatment regimen, eyes followed under the Pro ReNata regimen showed better sensitivity (100%) and specificity (93.3%) than eyes followed under the treat and extend regimen (93.5% and 88.6%, respectively). Based on the findings of this study, the follow-up for selected patients with nAMD under anti-VEGF treatment could be monitored without regular VA testing with acceptable performance.

## 1. Introduction

Neovascular age-related macular degeneration (nAMD) is the most common cause of blindness among the elderly in developed countries [1]. The disease is associated with an upregulation of vascular endothelial growth factor (VEGF) resulting in alteration of the blood–retinal barrier, causing extravasation of fluid into the extracellular space that appears clinically as macular edema, leading to visual loss [2]. Currently, intravitreal injections of anti-VEGF agents, ranibizumab (Lucentis^®^, Novartis Pharma AG, Basel, Switzerland) and aflibercept (Eylea^®^, Bayer, Barmen, Germany), are considered the standard of care for treating nAMD [3,4,5], and act by reducing the macular edema caused by the neovascular membrane.

Optical coherence tomography (OCT) is a fast, noninvasive technology that produces in vivo images of the retina, showing the presence or not of macular edema. OCT has become the most frequently used diagnostic tool in ophthalmology and has revolutionized clinical imaging for diagnosis and disease management in most retinal diseases including nAMD [6]. Currently, the combined analysis of OCT images and visual acuity (VA) measurements is considered to be the gold standard for follow-up and retreatment decisions. However, measuring VA is time-consuming, particularly in the elderly population. It is also considered subjective because it can fluctuate depending on the patient’s level of fatigue even in cases of non-active disease.

To our knowledge, few studies have investigated the role of VA in defining the retreatment strategy, while OCT seems essential to retinal disease management. OCT appears useful regardless of VA for determining when retreatment with anti-VEGF is necessary in nAMD [7]. This observation is even more important since the outbreak of the COVID-19 pandemic when ophthalmology departments had to modify their treatment protocols: bilateral injections on the same day, fixed treatment regimens and canceling of VA measurement [8].

The aim of this study is to assess whether the retreatment decision in patients with nAMD could be determined based on anatomical criteria only (mostly OCT-guided), compared to the gold standard of anatomical and functional criteria (with addition of VA).

## 2. Materials and Methods

We performed a cross-sectional study from September 2021 to December 2021 including patients followed for nAMD within the ophthalmology tertiary retinal reference center at the Croix-Rousse University Hospital in Lyon, France. The inclusion criteria were age ≥ 50 years old, and the presence of a documented macular neovascularization (MNV) associated with nAMD, treated with anti-VEGF therapy at the time of inclusion. Patients with cataracts (if causing significant visual impairment), aphakia, vitreous hemorrhage, history of rhegmatogenous retinal detachment, uncontrolled glaucoma, or another macular disease were excluded. This study was performed in accordance with the Declaration of Helsinki and current French legislation. The informed consent of the patients was obtained, and a local Ethics Committee (Hospices Civils de Lyon) approved the study registered under the number 20-156.

The following variables were assessed: VA, assessed in a seated position using an Early Treatment Diabetic Retinopathy Study (ETDRS) scale at an initial testing distance of 4 m or at 1 m (if not possible to assess at 4 m); the presence of retinal hemorrhage on ultra-wide-field retinography (Optos California system, Optos PLC, Dunfermline, Scotland, UK); OCT images were taken using a spectral-domain OCT (SD-OCT) device (HRA Spectralis, Heidelberg Engineering, Heidelberg, Germany); central retinal thickness (CRT) was assessed as well as qualitative parameters: intraretinal fluid (IRF), subretinal fluid (SRF) or subretinal hyperreflective material (SHRM). The MNV subtype was also defined using multimodal imaging including fluorescein angiography and indocyanine green angiography (HRA Spectralis, Heidelberg Engineering, Heidelberg, Germany). The other parameters retrieved were as follows: duration of the VA measurement, the anti-VEGF molecule used, treatment regimen, duration of follow-up and treatment, number of intravitreal injections and the results of the dilated fundus examination.

At inclusion, an initial therapeutic decision was made based on the analysis of the SD-OCT by senior retina specialists with no knowledge of the results of the VA measurement (OCT-guided strategy). In a second step, this decision was maintained or modified after being made aware of the VA measurement: in the case of a pro re nata (PRN) protocol, the decision was to proceed with treatment or simple monitoring; in the case of a treat and extend (TAE) protocol: the decision was to maintain, extend or reduce the retreatment period. Two retinal specialists (B.E.A and M.C) discussed the retreatment decision, and in case of disagreement, a third senior specialist (L.K) decided if the eye needed retreatment or not. Before decision of treatment, the retinal specialists had access to the retinography and the OCT of the day, and to all previous data in terms of VA measurement, OCT imaging and retinography. Secondarily, the VA testing of the day was revealed, and the retinal specialists could change their decision of retreatment. This methodology allowed the assessment of the added value of VA testing in the retreatment decision.

As an observational real-life study, the treatment regimen and the retreatment decision were made at the ophthalmologists’ discretion. However, in our retinal tertiary center, international guidelines from randomized controlled trials, were generally used to define the need for retreatment. On OCT, treatment was generally decided when active exudation was observed: presence of SRF, unless stable (i.e., persistent) since the last 3 monthly injections, presence of IRF or increased in CMT, presence of SHRM. On visual acuity testing, a loss of ≥ 5 letters since the previous visit, with no subretinal atrophy or fibrosis, also indicated a retreatment.

All data collected from patients were entered anonymously into an Excel spreadsheet (Microsoft Corp., Redmond, WA, USA) and processed using XLSTAT statistical software (Addinsoft, New York, NY, USA). Analyses of correlation between CRT and the VA measurement were performed using Spearman’s test. Sensitivity and specificity analyses of the OCT-guided strategy versus the gold standard (OCT and VA) were calculated. The information obtained by comparing OCT-guided strategy with the gold standard was conventionally summarized in a two-by-two table. The true positives are the patients in whom the OCT-guided strategy led to a decision to inject (for PRN regimen) or to reduce/maintain the interval (for TAE regimen) and for whom this decision was confirmed by the gold standard. True negatives are patients in whom OCT-guided strategy led to a decision not to inject (for PRN regimen) or to extend the interval (for TAE regimen) and for whom this decision was confirmed by the gold standard. False positives are patients in whom OCT-guided strategy led to the decision to perform an injection or reduce/maintain the interval, but who were not injected after obtaining the visual acuity results. False negatives are patients in whom OCT-guided strategy led to the decision not to perform an injection or to extend the interval, but who ultimately were injected after obtaining the visual acuity results. A *p*-value < 0.05 was considered statistically significant.

## 3. Results

### 3.1. Patient Characteristics

A total of 142 eyes in 126 patients with nAMD were included in the present study. The mean (SD) duration of follow-up was 4.2 (2.0) years between the onset of disease and patient inclusion, and the mean (SD) number of injections was 14.9 (6.3). Mean (SD) VA was 63.4 (22.2) letters at the onset of disease and 62.1 (23.5) letters at the time of inclusion. Mean (SD) CRT was 300.9 (76.2) µm. No retinal hemorrhage was observed on the fundus for any patients (Table 1).

### 3.2. Treatment Decision Change with the Addition of VA

**Figure 1:**
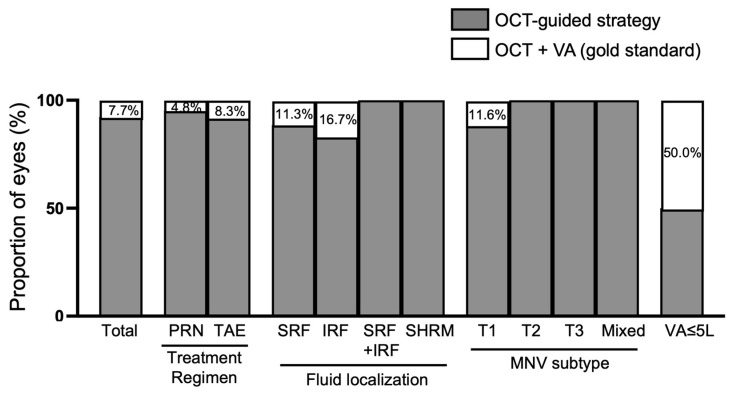
Performance of OCT-guided strategy for retreatment decisions in comparison to the gold standard (visual acuity + OCT). IRF: intraretinal fluid; MNV: macular neovascularization; OCT: optical coherence tomography; PRN: pro re nata; SHRM: subretinal hyperreflective material; SRF: subretinal fluid; TAE: treat and extend; T1-T2-T3: type 1-2-3; VA: visual acuity.

In the whole population, the retreatment decision based on anatomical criteria only (mostly OCT-guided) was same as the gold standard (i.e., OCT + VA) in 92.3% of cases. In the 11 cases (7.7%) where the addition of VA changed the retreatment decision, 6 eyes (4.2%) were finally not reinjected (PRN) or treatment was extended (TAE), and 5 eyes (3.5%) were reinjected (PRN) or treatment was shortened (TAE). Considering the specific subgroup analyses, adding VA to OCT led to different retreatment decision outcomes. According to the treatment regimen, the knowledge of VA changed the treatment decision in 4.8% of cases for PRN and 8.2% of cases for the TAE regimen. According to the fluid location, the knowledge of VA changed the treatment decision in 11.5% of cases for SRF only, 16.7% of cases for IRF only, 0% of cases for SRF + IRF and 0% of cases of SHRM only. According to MNV subtype, the knowledge of VA changed the OCT decision in 11.6% of cases for type 1 MNV, and in 0% for all other MNV subtypes. For late-stage nAMD (with VA ≤ 5 letters), the addition of VA changed the treatment decision in 50.0% of cases (Figure 1).

### 3.3. Sensitivity and Specificity of OCT-Guided Strategy in Comparison to OCT and VA

In the whole population, the OCT-guided strategy had a sensitivity of 94.0% (95%CI (86.2–97.7)) and specificity of 89.8% (95%CI (79.2–95.5)); the corresponding PPV and NPV were 92.9% (95%CI (87.3–98.4)) and 91.4% (95%CI (84.2–98.6)), respectively (Table 2).

Considering the treatment regimen, eyes followed under a PRN regimen showed better sensitivity and specificity than eyes followed under TAE. According to the MNV subtypes, type 1 MNV had the lowest sensitivity and specificity compared to the other subtypes, for which the OCT-guided strategy performed as well as the gold standard. According to VA, sensitivity and specificity was poor for eyes showing late-stage nAMD (VA ≤ 5 letters) (Table 3).

### 3.4. VA Measurement

**Figure 2:**
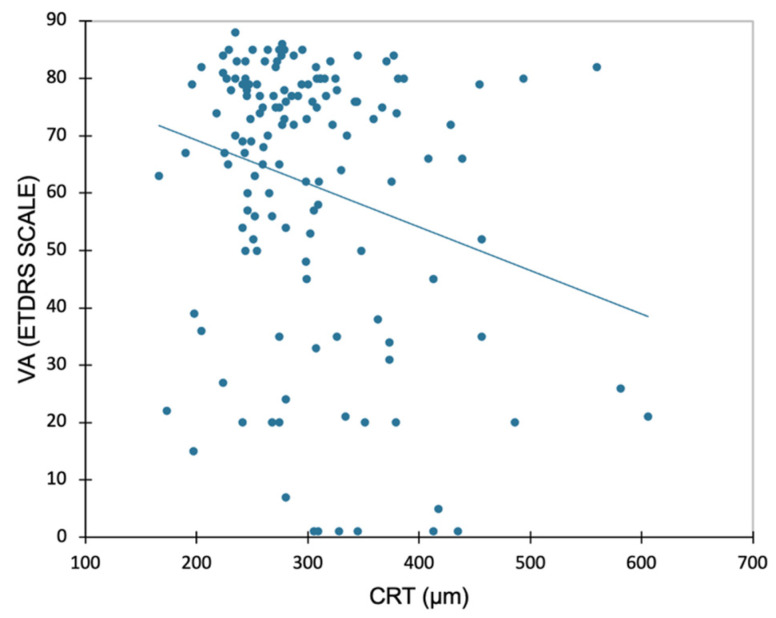
Linear regression plot for visual acuity (VA, ETDRS Scale) and central retinal thickness (CRT) at the study visit. Correlation analysis performed using Pearson’s test (r = −0.241; *p* = 0.004).

The mean (SD) duration of the visual acuity measurement was 6.2 (2.4) minutes. A significant but very weak negative association was found between VA and CRT, showing that CRT increases as VA decreases (r = −0.24; *p* = 0.004, Figure 2).

### 3.5. Disagreement between OCT and Gold Standard

In the 11 cases where the decision to retreat based on the OCT-guided strategy differed from the decision based on the gold standard of VA + OCT, 6/11 eyes were not reinjected after VA was revealed and were considered false positives. These mismatches were explained by the presence of persistent fluid (with no IRF or SRF fluctuations) with no VA loss in 5/6 cases, or late-stage AMD with no functional treatment effectiveness in 1/6 case. Moreover, 5/11 other eyes were finally reinjected after VA was revealed and were considered false negatives. These mismatches were explained by late-stage AMD with visual loss in 3/5 cases, and the presence of persistent fluid (with no IRF or SRF fluctuations) with VA loss in 2/5 cases.

## 4. Discussion

In the present study, the retreatment decision was generally well-guided based on the anatomical criteria alone, with a sensitivity of 94% and a specificity of almost 90%. Adding VA to anatomical criteria changed the therapeutic decision for less than 10% of patients. However, it should be noted that our population included patients already treated for nAMD and followed in a tertiary retinal center whose vision was not expected to change significantly between two appointments. The PPV indicates that nearly 93% of eyes for which OCT indicated the need for intravitreal injection were effectively treated after VA was revealed. Nevertheless, the remaining 7% (six eyes) would have been retreated based on the OCT-guided strategy, although they ultimately were shown not to need retreatment after the VA measurements. At the same time, the NPV indicates that nearly 91% of eyes for which the OCT-guided strategy did not indicate the need for intravitreal injection were not retreated in light of the VA results. Nevertheless, the remaining 9% (five eyes) would not have been retreated if the decision was based on the OCT-guided strategy, although the VA measurements showed treatment was required. These results are in agreement with the literature where OCT appears useful for determining when retreatment with anti-VEGF is necessary [7], but to our knowledge, no study assessing the performance of an OCT-guided strategy when making retreatment decisions has been carried out to date. VA measurement is time consuming, especially because of the advanced age of nAMD patients which means they take longer to respond and move around. The duration of VA measurement only (with no movement) is estimated at 6 min per patient. Moreover, it is well known that VA can change between two tests, depending on the test conditions, including the diligence of the VA examiner, and the patient’s level of tiredness, which is why the threshold of at least five letters is required to be clinically significant in standardized clinical studies [9]. In our study, a significant, but weak negative correlation between VA and CRT was found, showing that VA loss is generally associated with an increase in CRT. These findings demonstrate the predominant role of OCT in monitoring disease activity and progression, supported by the analysis of the evolution in the quantitative criteria (CRT) visualized on the mapping, and the qualitative assessment of fluid [6,10,11]. The PrONTO study used an OCT-guided variable-dosing regimen with intravitreal ranibizumab resulting in VA outcomes comparable to those seen with monthly dosing, but fewer intravitreal injections were required [12]. Conversely, a prospective study analyzed the performance of VA alone for retreatment decisions in nAMD treated with ranibizumab. Patients were randomized in two groups: retreatment guided by VA loss; or VA loss and/or signs of disease activity on OCT. The study was closed prematurely at one year (instead of two years follow-up) because of the updated label for ranibizumab. However, the intermediate analysis of outcome measures at one year showed numerically better VA in the group assessed using VA and OCT [13]. It is now well demonstrated in nAMD that VA loss can be preceded by anatomical changes observed on OCT [14] and even more recently on OCT-Angiography [15], and therefore, it could be used as an early indicator for retreatment decisions. These results are confirmed by the guidelines for the management of nAMD, reporting that VA measurement alone is insufficient to detect a recurrence of activity in the neovascular membranes [10].

These studies show that the qualitative criteria, in addition to the quantitative criteria (CRT), on the OCT images are important to the therapeutic decision. The presence or absence of disease activity (i.e., presence of fluid) on OCT could therefore guide treatment decisions in AMD. However, depending on the presence of fluids and their localization, OCT-guided retreatment decisions have led to different outcomes. Although in the presence of both SRF and IRF, or SHRM alone, the knowledge of VA in addition to OCT did not change the retreatment decision, in the presence of SRF or IRF alone, VA did change the decision in some cases. These mismatches between OCT-guided strategy and OCT + VA could be explained by rare cases of fluid persistence despite frequent anti-VEGF injections in cases where fluid is tolerated [16]. In a randomized controlled study, a relaxed TAE regimen, tolerating a small amount of fluid, demonstrated similar functional outcomes as a strict TAE regimen. This relaxed protocol could be applied to eyes presenting persistent SRF or IRF.

Nevertheless, there are some situations where VA may be relevant for treating nAMD patients. The most obvious case is late-stage nAMD with low vision (VA ≤ 5 letters). In such cases, the retina is generally dramatically damaged with fibro-atrophic scar and residual subretinal and/or intraretinal fluid visible on OCT. The retreatment decision is therefore guided by patients’ complaints of vision loss, whether this was reported on VA measurement or not. In the present study, the OCT-guided strategy matched the final therapeutic decision in only 50% of cases. Regarding the MNV subtypes, it should be noted that all, except type 1 MNV, could be managed with OCT-guided strategy. Type 1 MNV, previously described as occult choroidal neovascularization, is known to be the MNV subtype with the best prognosis, and a case in which subretinal fluid can be sometimes tolerated [16]. The knowledge of VA is therefore of importance and can reveal poorer tolerance of this residual fluid, prompting intravitreal anti-VEGF therapy. Analyzing our results and the descriptions of patients for whom the OCT-guided retreatment decision did not match the gold standard shows that the false positive are mostly patients with persistent fluid, and the false negatives mostly patients with late-stage AMD, confirming the above hypotheses.

Herein, no retinal hemorrhage was observed on retinography for any patients, therefore preventing any conclusions for the systematical use of fundus examination in treatment decision in our study. Patel and al. found that ranibizumab retreatment guided by monthly SD-OCT achieved similar vision gains with or without injection in the presence of hemorrhage without OCT-detectable fluid. These findings suggest that macular hemorrhage with no OCT-detectable macula fluid may not require treatment [17]. Other studies found similar patient outcomes with or without a dilated fundus. This suggests that dilated fundus may not be needed at every visit and nAMD patients could be followed up relying on imaging alone to make retreatment decisions, thereby reducing the time spent by patients in hospital [18]. However, the exact localization of macular hemorrhage within the retinal layers and in the macular area needs to be specified, as superficial, deep intraretinal or subretinal hemorrhage have different implications for visual prognosis. Moreover, macular hemorrhage outside the OCT image coverage but close to the fovea may have a risk of spreading rapidly to the foveal region and irreversibly affecting VA. Therefore, retinal photography or dilated fundus could be regularly realized, especially if the patient reported ocular symptoms (scotoma, loss of vision…).

This study has several limitations. Firstly, the monocentric design of the study is not necessarily representative of the worldwide management of nAMD patients. However, it does ensure the harmonization of retreatment decisions according to the clinical scenario. Secondly, the observational nature of the study may induce bias in the standardization of the retreatment decisions. For instance, the retreatment decision for persistent fluid is not widely consensual and calls for several characteristics such as type of fluid, volume, chronicity…, therefore preventing the generalization of our results to all retinal centers. For such a case, the decline in VA despite no change in OCT features could be an indicator for retreatment. Finally, our cohort could be considered small when analyzing particular situations (i.e., subgroup analyses), but it does open up avenues for more specific future studies to explore.

This study suggests that the relevance of VA measurement should be assessed for each patient individually to reduce the time spent in clinic, thereby improving the quality of care and the comfort without affecting treatment performance. This result is all the more interesting in the context of the COVID-19 pandemic where older patients are most vulnerable, and it might be wise to keep them away from hospitals and standard patient flows.

## 5. Conclusions

Based on the findings of the present study, the follow-up for selected patients with nAMD under anti-VEGF treatment could be monitored without regular VA testing with acceptable performance. This suggests, on the one hand, that VA testing does not seem to be mandatory in the retreatment decision, except in some specific cases (late-stage nAMD, persistent fluid…), and on the other hand, that spacing VA measurement intervals may be considered in order to improve the quality of care and patient comfort by reducing the time spent in clinic. However, long-term studies are needed to verify if this strategy is still favorable in the whole follow-up of a patient with nAMD.

## Figures and Tables

**Table 1:** Demographics and disease characteristics at the study visit.

Gender, n (%)	
Men	42 (33.3%)
Women	84 (66.7%)
**Mean (SD) age, years**	81.5 (6.7)
**Laterality, *n* (%)**	
Right	85 (59.9%)
Left	57 (40.1%)
**Molecule, *n* (%)**	
Aflibercept	106 (74.6%)
Ranibizumab	36 (25.3%)
**Fluid localization, *n* (%)**	
SRF	27 (19.0%)
IRF	36 (25.4%)
SRF and IRF	5 (3.5%)
SHRM	4 (2.8%)
Absence of fluid	70 (49.3%)
**Macular neovascularization subtype, *n* (%)**	
Type 1	97 (68.3%)
Type 2	18 (12.7%)
Type 3	15 (10.6%)
Mixed	12 (8.4%)
**Treatment regimen, *n* (%)**	
PRN	21 (14.8%)
TAE	121 (85.2%)
**Mean time since first diagnosis of nAMD, years (SD)**	4.2 (2.0)
**Mean number of injections since first diagnosis, *n* (SD)**	14.9 (6.3)
**Mean VA at disease onset, ETDRS letters (SD)**	63.4 (22.2)
**Mean VA at study visit, ETDRS letters (SD)**	62.1 (23.5)
**Late-stage nAMD (VA ≤ 5 letters), *n* (%)**	8 (5.6)
**Mean CRT, µm (SD)**	300.9 (76.2)

CRT: central retinal thickness; ETDRS: early treatment diabetic retinopathy study; IRF: intraretinal fluid; nAMD: neovascular age-related macular degeneration; PRN: pro re nata; SD: standard deviation; SHRM: subretinal hyperreflective material; SRF: subretinal fluid; TAE: treat and extend; VA: visual acuity.

**Table 2:** Contingency table comparing optical coherence tomography (OCT)-guided strategy with the gold standard (OCT + visual acuity test), in the decision for retreatment with intravitreal injection (IVT) of anti-VEGF.

	IVT+ (*n* = 83)	IVT− (*n* = 59)		
**OCT+ (*n* = 84)**	78 (55.0)	6 (4.2)	⇛	Positive predictive value 92.9% (87.3–98.4)
**OCT− (*n* = 58)**	5 (3.5)	53 (37.3)	⇛	Negative predictive value 91.4% (84.2–98.6)
	⤋ Sensitivity 94.0% (86.2–97.7)	⤋ Specificity 89.8% (79.1–95.5)		

**Table 3:** Sensitivity and specificity of OCT-guided strategy for retreatment decisions in nAMD patients.

	Sensitivity	Specificity	PPV	NPV
Whole population	94.0% (86.2–97.7)	89.8% (79.2–95.5)	92.9% (87.3–98.4)	91.4% (84.2–98.6)
Treatment regimen PRN TAE	100% (55.2–100) 93.5% (85.2–97.5)	93.3% (67.8–100) 88.6% (75.4–95.4)	85.7% (59.8–100) 93.5% (88.0–99.0)	100% (100–100) 88.6% (79.3–98.0)
Macular neovascularization Type 1 Type 2 Type 3 Mixed	91.1% (80.2–96.5) 100% (67.4–100) 100% (62.2–100) 100% (59.0–100)	84.6% (69.8–93.0) 100% (62.2–100) 100% (59.0–100) 100% (50.6–100)	89.5% (81.5–97.4) 100% (100–100) 100% (100–100) 100% (100–100)	86.8% (76.1–97.6) 100% (100–100) 100% (100–100) 100% (100–100)
Late-stage nAMD (VA ≤ 5 letters)	50.0% (19.0–81.0)	50.0% (10.0–90.0)	75.0% (32.6–100)	25.0% (0–67.4)

nAMD: neovascular age-related macular degeneration; NPV: negative predictive value; PPV: positive predictive value; TAE: treat and extend; PRN: pro re nata; VA: visual acuity.

## Data Availability

Not applicable.
